# Supplementary material for: Patterns of metastases progression- The linear parallel ratio
Source: PLoS One. 2022 Sep 21;17(9):e0274942. doi: 10.1371/journal.pone.0274942 (PMC9491615; doi:10.1371/journal.pone.0274942)
Supplement: S1 File — (DOCX) [file pone.0274942.s001.docx]

EPSILON = 1

**def** calc_lpr(metastases_lst):
 *"""
 Calculates the LPR of the Metastases list* **:param** *metastases_lst: Sorted list of metastases sizes in mm. Assuming
 len(metastases_lst) > 0* **:return***: Linear Parallel Ratio of the Metastases list, as explained in
 the manuscript.
 """* clustered_count = 0
 isolated_count = 0
 current_met_group = [metastases_lst[0]]
 **for** met **in** metastases_lst[1:]:
 min_met_in_cur_group = min(current_met_group)
 *# this met belongs in current group* **if** met - min_met_in_cur_group <= EPSILON:
 current_met_group.append(met)
 **continue** *# met - min_met_in_cur_group > EPSILON therefore we need to start a
 # new group:* **else**:
 **if** len(current_met_group) == 1:
 isolated_count += 1
 **elif** len(current_met_group) >= 1:
 clustered_count += len(current_met_group)
 current_met_group.clear()
 current_met_group.append(met)
 *# Handle last group* **if** len(current_met_group) == 1:
 isolated_count += 1
 **elif** len(current_met_group) >= 1:
 clustered_count += len(current_met_group)

 **return** (clustered_count - isolated_count) / (clustered_count + isolated_count)
