# Supplementary material for: Patterns of metastases progression- The linear parallel ratio
Source: PLoS One. 2022 Sep 21;17(9):e0274942. doi: 10.1371/journal.pone.0274942 (PMC9491615; doi:10.1371/journal.pone.0274942)
Supplement: S1 Table — (DOCX) [file pone.0274942.s002.docx]

Table S1: Basic parameters of patients with lung metastases

| Primary  tumour | Number of patients | Average Age at diagnosis (SD) | Gender  F/M (%) | Metastasis present upon diagnosis (%) | Average time from diagnosis to metastases in months (SD)* |
| --- | --- | --- | --- | --- | --- |
| Thyroid | 27 | 62.1 (14.4) | 12/15 (44.4) | 19 (70.4) | 58.0 (74.8) |
| Pancreas | 30 | 67.7 (11.9) | 14/16 (46.7) | 28 (93.3) | 25.5 (12.0) |
| Prostate | 26 | 69.9 (10.8) | 0/26 (0) | 20 (76.9) | 63.8 (46.1) |
| Kidney | 45 | 64.7 (12.3) | 9/36 (20) | 17 (37.8) | 53.8 (50.6) |
| Melanoma | 48 | 60.2 (16.9) | 21/27 (43.8) | 18 (37.5) | 29.1 (24.4) |
| Colorectal | 149 | 61.1 (14.9) | 68/81 (45.6) | 90 (60.4) | 25.6 (18.6) |
| Breast | 72 | 56.8 (13.9) | 66/6 (91.7) | 42 (58.3) | 66.0 (62.9) |
| Bladder | 35 | 71.4 (8.3) | 4/31 (11.4) | 13 (37.1) | 27.9 (30.1) |
| Sarcomas | 71 | 50.6 (19.3) | 39/32 (54.9) | 33 (46.5) | 32.7 (50.8) |
| Overall | 503 | 60.8 (16.0) | 233/270 (46.3) | 280 (55.7) | 38.8 (44.6) |

*In patients presenting without metastases
