# Supplementary material for: Patterns of metastases progression- The linear parallel ratio
Source: PLoS One. 2022 Sep 21;17(9):e0274942. doi: 10.1371/journal.pone.0274942 (PMC9491615; doi:10.1371/journal.pone.0274942)
Supplement: S2 Table — (DOCX) [file pone.0274942.s003.docx]

**Table S2. Outcome of patients with lung metastases**

| Primary  tumour | Average follow-up in months (SD)* | Died (%) | Disease specific death (%) | 2-year overall survival |
| --- | --- | --- | --- | --- |
| Thyroid | 76.0 (83.5) | 17 (62.9) | 17 (100) | 79.2 |
| Pancreas | 12.9 (11.9) | 28 (93.3) | 28 (100) | 18.7 |
| Prostate | 67.0 (51.8) | 21 (80.7) | 20 (95.2) | 80.1 |
| Kidney | 56.2 (68.4) | 37 (82.2) | 35 (94.6) | 52.3 |
| Melanoma | 38.2 (35.7) | 38 (79.1) | 36 (94.7) | 56.1 |
| Colorectal | 43.6 (27.3) | 120 (80.5) | 119 (99.2) | 78.3 |
| Breast | 67.6 (63.6) | 62 (86.1) | 59 (95.2) | 76.8 |
| Bladder | 33.3 (34.5) | 32 (91.4) | 32 (100) | 45.8 |
| Sarcomas | 29.2 (42.3) | 52 (73.2) | 52 (100) | 62.0 |
| Overall | 47.4 (49.6) | 406 (80.7) | 401 (98.7) | 66.7 |

*From diagnosis to censoring
