# Supplementary material for: Patterns of metastases progression- The linear parallel ratio
Source: PLoS One. 2022 Sep 21;17(9):e0274942. doi: 10.1371/journal.pone.0274942 (PMC9491615; doi:10.1371/journal.pone.0274942)
Supplement: Attachment — Submitted filename: Response to Reviewers.docx [file pone.0274942.s005.docx]

Nikolas K. Haass, MD/PhD	23.06.2022
Academic Editor
PLOS ONE

Dear Editor

Thank you for your letter and for the reviewers’ comments regarding manuscript PONE-D-22-06443, Patterns of Metastases Progression- The Linear Parallel Ratio. We have addressed the reviewers’ comments below, and hope you find this manuscript suitable for publication in PLOS ONE.

**Editorial Comments:**

1. Please ensure that your manuscript meets PLOS ONE's style requirements, including those for file naming. The PLOS ONE style templates can be found at <https://journals.plos.org/plosone/s/file?id=wjVg/PLOSOne_formatting_sample_main_body.pdf> and <https://journals.plos.org/plosone/s/file?id=ba62/PLOSOne_formatting_sample_title_authors_affiliations.pdf>.

**Response:** This was corrected as requested.

2. Thank you for stating the following financial disclosure:

**Response:** This was corrected as requested.

At this time, please address the following queries:

a) Please clarify the sources of funding (financial or material support) for your study. List the grants or organizations that supported your study, including funding received from your institution.
b) State what role the funders took in the study. If the funders had no role in your study, please state: “The funders had no role in study design, data collection and analysis, decision to publish, or preparation of the manuscript.”
c) If any authors received a salary from any of your funders, please state which authors and which funders.
d) If you did not receive any funding for this study, please state: “The authors received no specific funding for this work.”

Please include your amended statements within your cover letter; we will change the online submission form on your behalf.

**Response:** This was done.

3. Thank you for stating the following in your Competing Interests section:  
No

Please complete your Competing Interests on the online submission form to state any Competing Interests. If you have no competing interests, please state "The authors have declared that no competing interests exist.", as detailed online in our guide for authors at <http://journals.plos.org/plosone/s/submit-now> 
 This information should be included in your cover letter; we will change the online submission form on your behalf.

**Response:** This was done.

4. Please include your full ethics statement in the ‘Methods’ section of your manuscript file. In your statement, please include the full name of the IRB or ethics committee who approved or waived your study, as well as whether or not you obtained informed written or verbal consent. If consent was waived for your study, please include this information in your statement as well.

**Response:** This was done.

5. Please include your tables as part of your main manuscript and remove the individual files. Please note that supplementary tables (should remain/ be uploaded) as separate "supporting information" files.

**Response:** This was done.

Additional Editor Comments:

I find your manuscript very interesting, as it provides some novel insights into metastasis and the interpretation of CT scans. In accordance with the expert reviewers, there are however, a few concerns that need to be addressed. Rather than repeat those points here, I refer you to the specific remarks (below) for details.

**Response:** I wish to thank the editor.

**Reviewers' comments:**

**Comments to the Author**

1. Is the manuscript technically sound, and do the data support the conclusions?

The manuscript must describe a technically sound piece of scientific research with data that supports the conclusions. Experiments must have been conducted rigorously, with appropriate controls, replication, and sample sizes. The conclusions must be drawn appropriately based on the data presented.

Reviewer #1: Yes

Reviewer #2: Partly

 2. Has the statistical analysis been performed appropriately and rigorously?

 Reviewer #1: N/A

Reviewer #2: Yes

 3. Have the authors made all data underlying the findings in their manuscript fully available?

The [PLOS Data policy](http://www.plosone.org/static/policies.action#sharing) requires authors to make all data underlying the findings described in their manuscript fully available without restriction, with rare exception (please refer to the Data Availability Statement in the manuscript PDF file). The data should be provided as part of the manuscript or its supporting information or deposited to a public repository. For example, in addition to summary statistics, the data points behind means, medians and variance measures should be available. If there are restrictions on publicly sharing data—e.g., participant privacy or use of data from a third party—those must be specified.

 Reviewer #1: Yes

Reviewer #2: Yes

 4. Is the manuscript presented in an intelligible fashion and written in standard English?

PLOS ONE does not copyedit accepted manuscripts, so the language in submitted articles must be clear, correct, and unambiguous. Any typographical or grammatical errors should be corrected at revision, so please note any specific errors here.

 Reviewer #1: Yes

Reviewer #2: Yes

5. Review Comments to the Author

Please use the space provided to explain your answers to the questions above. You may also include additional comments for the author, including concerns about dual publication, research ethics, or publication ethics. (Please upload your review as an attachment if it exceeds 20,000 characters)

 Reviewer #1: The authors present a study examining CT features to trace the lineage of lung metastases. By measuring and comparing the size of lung mets in each patient, they draw conclusions regarding the metastatic progression of each of the 9 diseases included in the study. Some cancer types had a high linear/parallel ratio (LPR) that indicated similarity between the different metastases, while other cancers had low LPR indicating greater variation.

Overall, I find their observations interesting. These results could have clinical relevance as CT scans are routine in the diagnosis and treatment of cancer patients.

I have a few points that I’d like the authors to comment on:

1. For some of the cancers presented here, melanoma and breast cancer for example, there are multiple histological subtypes that are related to different treatments and varied patient outcomes. Perhaps the data is oversimplified by combining the subtypes. Can you be more specific about the clinical data presented. Is some of the variation in LPR in theses cancers due to histological subtype?

**Response:** We agree with the reviewer. The model suggested is probably oversimplified (as almost any model in biology). This was added to the discussion (second paragraph, page 2). This is even more pronounced in the case of thyroid cancer, which includes both the good prognosis papillary carcinoma and the poor prognosis anaplastic carcinoma. Still, all the subjects included in this study had lung metastases and thus represent the topmost aggressive forms of the disease, a feature that can potentially decrease the variability in the LPR. The variability in the LPR was found to be associated with the LPR level itself (Fig. 4); tumors with high LPR (pancreas, prostate, and thyroid) had a low variability and tumors with low LPR (bladder, sarcoma and melanoma) had high variability. This point is mentioned at the end of the second paragraph of the Discussion section. Studying the LPR of different tumor subtypes is of interest and I hope this will take place.

2. Is there any relationship between LPR and treatment using targeted or immunotherapy?

**Response:** This interesting question was not examined in the current study.

3. Have you looked for a relationship between LPR and overall patient survival?

**Response:** LPR could be a prognostic factor, but this was not assessed in the current study.

4. Does the LPR for each cancer type align with published tumour mutation burden rates for each cancer?

**Response:** Some interesting findings were identified when comparing LPR with tumor mutation burden (TMB). Thyroid cancer with high LPR has a low TMB and melanoma with low LPR has a high TMB. Pancreatic tumors, on the other hand, have high LPR and high TMB. As a whole, correlation between LPR and TMB was intermediate (R^2^=0.024. p=0.6878), so no definite conclusion could be drawn.

| Primary | Median LPR | Median TMB* |
| --- | --- | --- |
| Thyroid | 1 | 16 |
| Pancreas | 1 | 187 |
| Prostate | 1 | 35 |
| Kidney | 0.91 | 63 |
| Melanoma | 0.65 | 276 |
| Colorectal | 0.6 | 74 |
| Breast | 0.58 | 32 |
| Bladder | 0.5 | 163 |
| Sarcomas | 0.43 | 58** |

*Castle JC et al. Mutation-Derived Neoantigens for Cancer Immunotherapy. Front Immunol. 2019;10:1856.

**Including uterine carcinosarcoma only

**Reviewer #2: Review**

This is an interesting study that reports on data obtained from lung CT scans.

Overall, I think it is worth publication in this journal. However, I there are a few issues that I believe the authors should address. Allow me to expand
as follows:

1. It is not clear the authors have a hypothesis even though they state they are performing the study to test their hypothesis. What is the hypothesis?
If there is one, it needs to be stated clearly, and it needs to be stated how these data obtained can address the likelihood of the hypothesis being true.
If this is not possible, then I think the study is best framed as an investigation that provides evidence that supports the hypothesis that
alternative metastatic disease morphologic patterns in lung (and by extension, possibly elsewhere) may reflect alternate pathogenic routes.
And the evidence is the correlation between known genomic data and CT morphologic patterns. Because there is a possible mechanism that may
account for this correlation, it may represent causation, thus a valid hypothesis may exist.

**Response:** The current study is based on the two leading models of metastatic progression, namely the linear and the parallel models (references 7-9). The hypothesis of the study is that they can be differentiated by measuring the diameters of metastases in CT images. This was stated more clearly in the last paragraph of page 3.

2. The authors provide evidence for such a correlation, but I think this should be the main finding of the investigation and should be part of the
abstract and conclusion. The review of the literature with respect to genomic studies in primary and secondary tumours is welcome. However,
any evidence that doesn’t support the idea that (i) uniformly size metastatic lesions are associated with genomic similarity (ii) variably sized metastatic lesions are associated with genomic dissimilarity (with respect to each tumour type) should also be reported. The readers can then weigh the evidence with respect to the hypothesis noted in #1.

**Response:** The study is based on the assumptions that with identical clonal origin and similar conditions within the host’s organ, similar growth kinetics are expected (page 4, 1^st^ paragraph). However, there is no direct evidence in the literature supporting the concept that genomic similarity equals uniform size. To the best of our knowledge, this issue has not been investigated yet, and will hopefully be studied after the current publication.

3. However, the most important question remains: how do metastases arise? It is an extraordinarily complex issue, and as the authors rightly point out, there are many potential confounders that prevent definitive conclusions to be drawn from morphologic data obtained from CT scans with respect to pathogenesis.
Although the study provides evidence associating morphologic patterns to genomic data, the derivation of the pathogenic route of metastatic disease
from genomic data alone, may not, in principle, be possible. For example, inter-tumour metastatic pleomorphism does not necessarily imply early and
pre-clinical seeding events, given the presence of significant genomic instability in many advanced tumours. The authors, I believe, should discuss
these issues in a little more detail.

**Response:** We agree with the reviewer. As mentioned in the first paragraph of the Discussion, the complexity of the metastatic process is high and includes early spread of DTCs, repeated waves of metastases, metastasis-to-metastasis spread, periods of dormancy, different growth rates of polyclonal cells etc. The dichotomy to linear and parallel routs is oversimplified as was discussed in response to comment 1 of reviewer 1 ("Introduction" second paragraph, page 2).

4. Although the ratio measured by the authors (LPR) seems reasonable, it is interesting that the lowest mean values are ~0.5. This is clearly
much larger than the potential lowest value of -1. Does this imply that the linear pathogenic route is ubiquitous? If there is an element of a parallel
pathogenic route in most tumours, how do we explain - from a biological perspective - both routes arising from the same tumour?

**Response:** We agree with the reviewer. The implication of the results is that the classical linear route is a more common pathway of spread. These results conform with most of the genomic studies of the various tumors, as reviewed in the Discussion section.

5. While the LPR has the advantage of being simple, are there more complicated measures that could be applied to the patient cohort that may
have added utility? For example, could the longest and shortest axis length of the metastatic tumours be pooled (with respect to the tissue of origin) and an unsupervised clustering algorithm applied to these data? Investigators could then simultaneously investigate tumour size and similarity of size. Perhaps different tumour types cluster into specific regions of axis-size space, with varying sizes of the resultant clusters - we might hypothesize that, for example, metastatic pancreatic tumours cluster into very few clusters, in a particular region of the space, with negligible intracluster variation. Perhaps the authors, in the discussion, could expand a little on the potential of
analyzing metastatic patterning in human cancer.

**Response:** The current study is an initial attempt to deduce the biology of the metastatic process purely from its morphology. As such, the easiest measurable parameter was used (long axis of each metastasis on CT axial images). As the reviewer suggests, better predictive parameters (such as, short axis, multiplication of the axes or perhaps metastases volume) could exist, and this point was added to the discussion (point 8 in the "limitations of the study" paragraph). I hope this research will draw attention to the morphology/biology association and that additional and possibly better parameters will be suggested.

 6. PLOS authors have the option to publish the peer review history of their article ([what does this mean?](https://journals.plos.org/plosone/s/editorial-and-peer-review-process#loc-peer-review-history)). If published, this will include your full peer review and any attached files.

If you choose “no”, your identity will remain anonymous but your review may still be made public.

**Do you want your identity to be public for this peer review?** For information about this choice, including consent withdrawal, please see our [Privacy Policy](https://www.plos.org/privacy-policy).

 Reviewer #1: No

Reviewer #2: No

 [NOTE: If reviewer comments were submitted as an attachment file, they will be attached to this email and accessible via the submission site. Please log into your account, locate the manuscript record, and check for the action link "View Attachments". If this link does not appear, there are no attachment files.]

While revising your submission, please upload your figure files to the Preflight Analysis and Conversion Engine (PACE) digital diagnostic tool, <https://pacev2.apexcovantage.com/>. PACE helps ensure that figures meet PLOS requirements. To use PACE, you must first register as a user. Registration is free. Then, login and navigate to the UPLOAD tab, where you will find detailed instructions on how to use the tool. If you encounter any issues or have any questions when using PACE, please email PLOS at [figures@plos.org](mailto:figures@plos.org). Please note that Supporting Information files do not need this step.
