# Supplementary material for: Patterns of metastases progression- The linear parallel ratio
Source: PLoS One. 2022 Sep 21;17(9):e0274942. doi: 10.1371/journal.pone.0274942 (PMC9491615; doi:10.1371/journal.pone.0274942)
Supplement: Attachment — Submitted filename: Response to Reviewers-2.docx [file pone.0274942.s006.docx]

Nikolas K. Haass, MD/PhD	6.08.2022
Academic Editor
PLOS ONE

Dear Editor

Thank you for your letter and for the thoughtful reviewer comment regarding manuscript PONE-D-22-06443, Patterns of Metastases Progression- The Linear Parallel Ratio. The response to the reviewer comment is below. Minor modifications were made in the manuscript. I hope that after this clarification you will find the manuscript suitable for publication in PLOS ONE.

Ofer Gofrit

**Reviewer comment:**

6. Review Comments to the Author

Please use the space provided to explain your answers to the questions above. You may also include additional comments for the author, including concerns about dual publication, research ethics, or publication ethics. (Please upload your review as an attachment if it exceeds 20,000 characters)

Reviewer #2: I still have an issue with the ideas regarding the hypothesis of this paper.

The authors state that the hypothesis is that the two leading models of metastatic progression can be identified by measuring the diameters of metastases (with the assumption that the leading models of tumour progression are, in fact, true - this latter point needs to made clear). But then how do these data support this hypothesis?

What would the expected distribution of metastatic sizes look like (between and within tumour types) if the leading models of metastatic spread were true? Or false? What if all tumours spread via a mixture of both mechanisms with differences in the variances (with respect to tumour type) of the tumour sizes? And how would that differ if the leading models of metastatic spread were mutually exclusive?

Could the results be simply due to stochastic variation where the two leading models of metastatic progression are irrelevant, or could the results be due to a mixture of both mechanisms of metastatic spread in all tumour types with the main differences between tumour types inherent and history-independent differences in the variances of the tumour sizes?

The problem is somewhat Bayesian. The authors are (sort of) asking Pr(A/B) where A is these data and B is the leading model of tumour spread, but the important quantity is Pr(B/A); that is, the probability of the theory of tumour spread being true given these data. Can the authors provide more rigour in the analysis of their data?

**Response:**

The metastatic process is extremely complex, and several models attempt to describe it (1^st^ paragraph of the “Discussion”). The leading models are the linear and the parallel models and they are supported by robust molecular data (ref 7-9). As any model in biology, they must be oversimplified, but they do offer a working framework that can be used in research and in the clinic.

In the current study we do not attempt to prove or disprove these models. We accept them as a basis for the study. The hypothesis of this work (stated in paragraph 5 in the ”Introduction”) is that ”differentiation between the linear and parallel routes of metastatic spread can be done by measurements of metastases diameters on the clinical CT”. We tested this hypothesis on nine tumors that commonly metastasize to the lungs and the results agree with molecular information obtained in complex and expensive experiments.

The data presented in this manuscript can potentially change our interpretation of CT scans without any additional cost. As mentioned in the “Discussion” the computer code, used in this research, is not necessary in the clinical work since the naked eye is sensitive to this type of information. For example, I saw in the clinic a few days ago a man with lung metastases from gall-bladder cancer (he came to me with urinary retention). His chest CT showed multiple metastases, but it was clear that they could all be fitted into two diameters only. This can be translated to LPR of one with two waves of metastases. As mentioned in the manuscript, this understanding may be relevant in several issues in oncology as in the definition of the oligometastatic state and in management of the primary tumor in a patient with metastatic disease.
